# Time-of-Day- and Light-Dependent Expression of Ubiquitin Protein Ligase E3 Component N-Recognin 4 (UBR4) in the Suprachiasmatic Nucleus Circadian Clock

**DOI:** 10.1371/journal.pone.0103103

**Published:** 2014-08-01

**Authors:** Harrod H. Ling, Christian Beaulé, Cheng-Kang Chiang, Ruijun Tian, Daniel Figeys, Hai-Ying M. Cheng

**Affiliations:** 1 Department of Biology, University of Toronto Mississauga, Mississauga, Ontario, Canada; 2 Ottawa Institute of Systems Biology and Department of Biochemistry, Microbiology and Immunology, University of Ottawa, Ottawa, Ontario, Canada; University of Texas Southwestern Medical Center, United States of America

## Abstract

Circadian rhythms of behavior and physiology are driven by the biological clock that operates endogenously but can also be entrained to the light-dark cycle of the environment. In mammals, the master circadian pacemaker is located in the suprachiasmatic nucleus (SCN), which is composed of individual cellular oscillators that are driven by a set of core clock genes interacting in transcriptional/translational feedback loops. Light signals can trigger molecular events in the SCN that ultimately impact on the phase of expression of core clock genes to reset the master pacemaker. While transcriptional regulation has received much attention in the field of circadian biology in the past, other mechanisms including targeted protein degradation likely contribute to the clock timing and entrainment process. In the present study, proteome-wide screens of the murine SCN led to the identification of ubiquitin protein ligase E3 component N-recognin 4 (UBR4), a novel E3 ubiquitin ligase component of the N-end rule pathway, as a time-of-day-dependent and light-inducible protein. The spatial and temporal expression pattern of UBR4 in the SCN was subsequently characterized by immunofluorescence microscopy. UBR4 is expressed across the entire rostrocaudal extent of the SCN in a time-of-day-dependent fashion. UBR4 is localized exclusively to arginine vasopressin (AVP)-expressing neurons of the SCN shell. Upon photic stimulation in the early subjective night, the number of UBR4-expressing cells within the SCN increases. This study is the first to identify a novel E3 ubiquitin ligase component, UBR4, in the murine SCN and to implicate the N-end rule degradation pathway as a potential player in regulating core clock mechanisms and photic entrainment.

## Introduction

Mammals possess numerous peripheral clocks throughout the body that keep track of time and control rhythmic outputs of behavior and physiology [1]. These endogenous peripheral clocks are temporally coordinated by a central pacemaker located in the suprachiasmatic nucleus (SCN), a hypothalamic structure directly above the optic chiasm [2], [3]. The SCN is a network of cellular oscillators that maintain molecular rhythms of core clock gene expression through the use of autoregulatory transcriptional/translational feedback loops (TTFLs) [4]. In addition, TTFLs are regulated by a wide array of post-transcriptional and post-translational mechanisms such as phosphorylation and acetylation, which fine-tune the rhythmic expression of core clock genes [5]. As these molecular oscillations have an endogenous period that only approximates a 24 h day/night cycle, daily adjustments to the phase of the internal clock are required to align it precisely to the local environmental time. This process of clock resetting in response to environmental light cues is known as photic entrainment [6]. The retinohypothalamic tract (RHT) represents a direct path by which light information is transmitted from the retina to the SCN [7],[8], where a series of molecular and cellular events are triggered that ultimately lead to transcription of immediate early genes and core clock genes [9] and thus resetting of the master pacemaker.

Beyond transcriptional control, other novel mechanisms are likely to contribute to core clock regulation and the entrainment process. One such mechanism is regulated protein degradation through the ubiquitin-proteasome pathway, which uses a series of enzymes, termed E1 (ubiquitin activating enzyme), E2 (ubiquitin conjugating enzyme) and E3 (ubiquitin ligase), to mark proteins intended for destruction with polyubiquitin tags [10]. The tagged protein is then recognized by the 26S proteome for degradation. Specific components of E3 ligase complexes have been implicated in mammalian core timing processes. For instance, the F-box protein, FBXL3, mediates the targeted destruction of cryptochrome (CRY) proteins [11]. As a result, mice with mutation of FBXL3 (the *overtime* and *after-hours* mutation) exhibit an abnormally long circadian period of 26–27 h [12], [13]. Another F-box protein, FBXL21, functions antagonistically to FBXL3 to regulate nuclear CRY protein turnover [14], [15]. Unlike FBXL3, loss of FBXL21 results in circadian period shortening [15]. Two E3 substrate receptors, β-TRCP1 and β-TRCP2, have also been shown to interact with PERIOD2 and affect its stability *in vitro* [16], [17]. In terms of a role in photic entrainment, proteasome-mediated degradation of the *Drosophila* clock protein, TIMELESS (TIM), is triggered by light and is required for photic resetting [18]. This light-induced TIM degradation involves ubiquitination of TIM with the help of the F-box protein, JETLAG (JET) [19]. Despite evidence for the involvement of specific E3 ligase complexes in core timing processes, the link between the ubiquitin-proteasome system and the entrainment process in mammals remains to be fully explored.

In an effort to understand better the molecular mechanisms that regulate core clock timing and photic entrainment, we recently conducted two separate proteome-wide screens of the murine SCN. One screen sought to identify proteins whose expression fluctuated as a function of time-of-day (Chiang et al., manuscript describing mass spectrometry screen was submitted). The second screen identified proteins that were differentially expressed after photic stimulation in the early night [20]. From these two screens emerged a novel candidate of clock- and light-dependent regulation: ubiquitin protein ligase E3 component N-recognin 4 (UBR4), a member of a special ubiquitin-dependent proteolytic system known as the ‘N-end rule pathway’ [21]. The current study characterizes the spatiotemporal expression and light responsiveness of UBR4 in the SCN. Our results reveal that UBR4 is expressed in a time-of-day-dependent fashion in AVP neurons of the SCN throughout its rostrocaudal extent. Furthermore, light signals in the early subjective night lead to an increase in the number of UBR4-expressing cells in this subset of SCN neurons.

## Materials and Methods

### Animals

Eight- to ten-week-old C57BL/6J male mice (The Jackson Laboratory, Bar Harbor, ME, USA) were used. Mice were group-housed (up to 5 animals per cage) in polycarbonate cages with *ad libitum* access to food and water throughout the experiment. All procedures followed the guidelines of the Canadian Council on Animal Care (http://www.ccac.ca) and were approved by the Local Animal Care Committees at the University of Ottawa (Ottawa, ON, Canada) and the University of Toronto Mississauga (Mississauga, ON, Canada). Cages were placed into a ventilated light-tight chamber with computer-controlled light schedules (Phenome Technologies, Chicago, IL, USA).

### Light Treatment Paradigm and Tissue Processing

Mice were entrained to a 12 h-light:12 h-dark cycle (100 lux intensity) for two weeks, and transferred to constant darkness (DD) for 2 cycles (48 h) prior to tissue harvest, with or without a preceding light pulse. For mass spectrometric (MS) analysis, mice were sacrificed on day 3 of DD at 4 h intervals at CT 2, 6, 10, 14, 18 and 22. To analyze circadian expression of UBR4 using indirect immunofluorescence (IF), DD mice were kept in conditions identical to those used for MS analysis, but were killed at 3 h intervals at CT 1, 4, 7, 10, 13, 16, 19 and 22 on day 3 of DD. To analyze light-inducible expression of UBR4, mice received a single light pulse (15 min, 100 lux) at CT 6, 15 or 22 on day 3 of DD, and killed 4 h later for tissue harvest. For each time point, dark controls were killed at the same time as the LP group. Data for UBR4 expression at CT19 and in dark controls for the CT15 LP group were obtained from the same set of mice. Tissues for CT6 LP and CT 22 LP groups (and their respective dark controls) were harvested, processed and stained in a separate experimental round from the CT15 LP group and corresponding dark control. For all experiments, CT was estimated based on the prior LD cycle, where Zeitgeber time (ZT) 12 (i.e. lights off) was used to define CT 12. N = 4 for each time point and treatment.

For tissue harvest, mice were killed by cervical dislocation and decapitated, and eyes were covered with black electrical tape under dim red light (using a red fluorescent light bulb and a Kodak series 2 filter [Eastman Kodak, Rochester, NY, USA], <5 lux at working distance of 1 meter from the light source). Brains were dissected, immersed in chilled oxygenation media, and sliced into 800-µm coronal sections containing the SCN with a vibratome. For Western blotting and MS analysis, the SCN was manually dissected from the coronal section using a razor blade, frozen immediately on dry ice, and stored at −80°C until use. For IF staining, coronal tissue sections were fixed in fresh 4% paraformaldehyde in phosphate-buffered saline (PBS), pH 7.4, for 4 h at 4°C on a rocker, and then transferred to 30% sucrose (w/v) in PBS overnight. Tissues were sliced into 30-µm thin sections on a freezing microtome and stored in 30% sucrose-PBS solution until staining.

### Cell Culture

For MS analysis, Neuro2A cells (American Type Culture Collection [ATCC], Manassas, VA) were grown in customized DMEM (AthenaES, Baltimore, MD, USA) supplemented with [^13^C_6_,^15^N_4_]-L-Arginine (Arg-10) and [^13^C_6_,^15^N_2_]-L-Lysine (Lys-8) (Sigma-Aldrich, Oakville, ON, Canada) at Arg 42 mg/L, Lys 146 mg/L, Met 30 mg/L, plus 10% (v/v) dialyzed FBS (Invitrogen, Burlington, ON, Canada), 1 mM sodium pyruvate (Invitrogen) and 28 µg/mL gentamicin (Invitrogen). Cells were maintained in culture for at least 10 doubling times to allow for complete (>98%) incorporation of the isotope-labeled amino acids. For UBR4 knockdown experiments, Neuro2A cells were cultured in regular growth medium (DMEM plus 10% FBS and penicillin-streptomycin), and transiently transfected with siRNA specific to UBR4 (ON-TARGET plus SMARTpool, Human ZUBR1, cat. no. L-014021-01-0005, ThermoFisher Scientific, Ottawa, ON, Canada) using Lipofectamine 2000 (Invitrogen) according to manufacturer's instructions.

### Western Blotting

SCN tissues from C57BL/6J mice were harvested and pooled (n = 2 mice) for subsequent protein extraction. Neuro2A cell lysates were harvested 24 h after transfection. Tissues and cell lysate were homogenized in ice-cold RIPA lysis buffer supplemented with a protease inhibitor cocktail. Proteins were quantified by Bradford protein assay using Coomassie Plus Protein Assay Reagent (Fisher Scientific, Ottawa, ON, Canada). Samples (40 µg) were resolved using 5% Tris-glycine SDS-PAGE, and blotted onto PVDF membranes by wet transfer using a buffer that was supplemented with 10% methanol and 0.005% SDS. The following primary antibodies were used: rabbit anti-UBR4 (1∶500; cat. no. HPA021046, Sigma-Aldrich, St. Louis, MO, USA) and rabbit anti-actin (1∶10,000; cat. no. A2066, Sigma-Aldrich). HRP-conjugated goat anti-rabbit IgG (1∶80,000; ThermoFisher Scientific) was also used. Signals were detected by chemiluminescence using the SuperSignal West Femto Maximum Sensitivity Substrate (ThermoFisher Scientific).

### Indirect Immunofluorescence

For most experiments, tissue slices from all animals were stained within the same session to minimize experimental variability. Brain slices were washed 5×5 min in PBS with 0.1% Triton X-100 (PBST), and blocked for 60 min at room temperature in PBST supplemented with 10% horse serum (PBST-HS). Sections were then transferred to a 1∶100 dilution of the rabbit anti-UBR4 antibody in PBST-HS and incubated overnight at 4°C on a shaker. Sections were washed 5×5 min in PBST and then incubated for 90 min at room temperature in a 1∶1000 dilution of an AlexaFluor 488 goat anti-rabbit secondary antibody (Invitrogen) in PBST-HS. Sections were washed 5×5 min in PBST, mounted onto microscope slides and coverslipped with fluorescence mounting medium (Dako S3023, Cedarlane, Burlington, ON, Canada). Sections from some animals were also double-stained using antibodies specific for arginine vasopressin (AVP) and vasoactive intestinal peptide (VIP). These sections were first stained for UBR4 as previously mentioned; after the washes following the secondary antibody, sections were blocked again in PBST-HS for 60 min at room temperature and transferred to a 1∶4000 dilution of a guinea pig anti-AVP primary antibody (Peninsula T-50548, Cedarlane) or 1∶3000 dilution of a guinea pig anti-VIP primary antibody (Peninsula T-5030, Cedarlane) in PBST-HS. Sections were incubated in the AVP/VIP primary antibody solution for 2 h at room temperature. Sections were washed 5×5 min in PBST and incubated in a 1∶1000 dilution of a DyLight 594 donkey anti-guinea pig IgG (Jackson Immunoresearch cat. no. 706-515-148, Cedarlane) for 90 min at room temperature. Sections were washed a final 5×5 min in PBST, mounted onto microscope slides and coverslipped with fluorescence mounting medium.

### Confocal Microscopy

Images were acquired using a Zeiss Axio Observer Z1 inverted microscope equipped with a Laser Scanning Microscope (LSM) 700 module and Zeiss LSM510 laser scanning confocal microscope (Carl Zeiss MicroImaging GmbH, Göttingen, Germany). Images were acquired with ZEN 2008 and ZEN 2010 software (Carl Zeiss MicroImaging GmbH). Low magnification images were obtained with a 20x objective (Plan-Apochromat 20X/0.8 M27 and EC Plan-Neofluar 20x/0.5) and high magnification images were obtained with a 63x oil immersion objective (Plan-Apochromat 63x/1.4). For 20X magnification, a Z-stack was acquired with 2.3-µm optical sections with 7 optical slices. Z-stacks of the same magnification were acquired with all confocal parameters (laser intensity, gain, pinhole size, scanning speed, and image averaging) held constant, in order to allow comparison between samples.

### Image Processing and Quantification

Confocal microscope images were processed and analyzed with Image J (Rasband, W.S., ImageJ, U. S. National Institutes of Health, Bethesda, Maryland, USA, http://imagej.nih.gov/ij/). When appropriate, each Z-stack was separated into individual acquisition channels (488 nm or 594 nm for UBR4 and AVP, respectively), and each channel was then re-stacked. For the 20x images, a maximum intensity projection of the entire Z-stack was produced of the SCN section for quantification. For each SCN at 20x, fluorescence intensity values for individual UBR4-expressing cells were obtained by measuring the grayscale intensity in a fixed circular shaped region of interest for each visible cell. Background of each SCN was obtained by measuring the grayscale intensity in a region of interest without any visible UBR4 cells. Mean normalized fluorescence intensity for each SCN section was calculated by subtracting the background intensity from mean of fluorescence intensity values for all cells measured. Numbers of UBR4-expressing cells in the SCN were also manually counted at 20x using maximum intensity projection of the Z-stack. Every cell whose immunofluorescent signal exceeded background staining was included in the count. Three sections were chosen per animal in each of the rostral, middle and caudal portion of the SCN for each time point analyzed. The mean normalized fluorescence intensity of individual cells and mean cell count of SCN were analyzed separately for the rostral, middle or caudal sections and mean values were computed and reported according to time point or treatment and sections.

### Statistical Analysis

Mean cell intensity and cell count for different CT were analyzed by a one-way ANOVA followed by LSD *post hoc* test. Changes in mean cell intensity and cell count of UBR4-expressing cells following light stimulation were analyzed by a two-way ANOVA with CT and light treatment as the independent variables. Significant interactions were explored with independent two-sample t-test with Bonferonni correction. Alpha was set at 0.05 for all statistical analyses.

## Results

### Mass Spectrometric Identification of UBR4 in the Murine SCN

**Figure 1:**
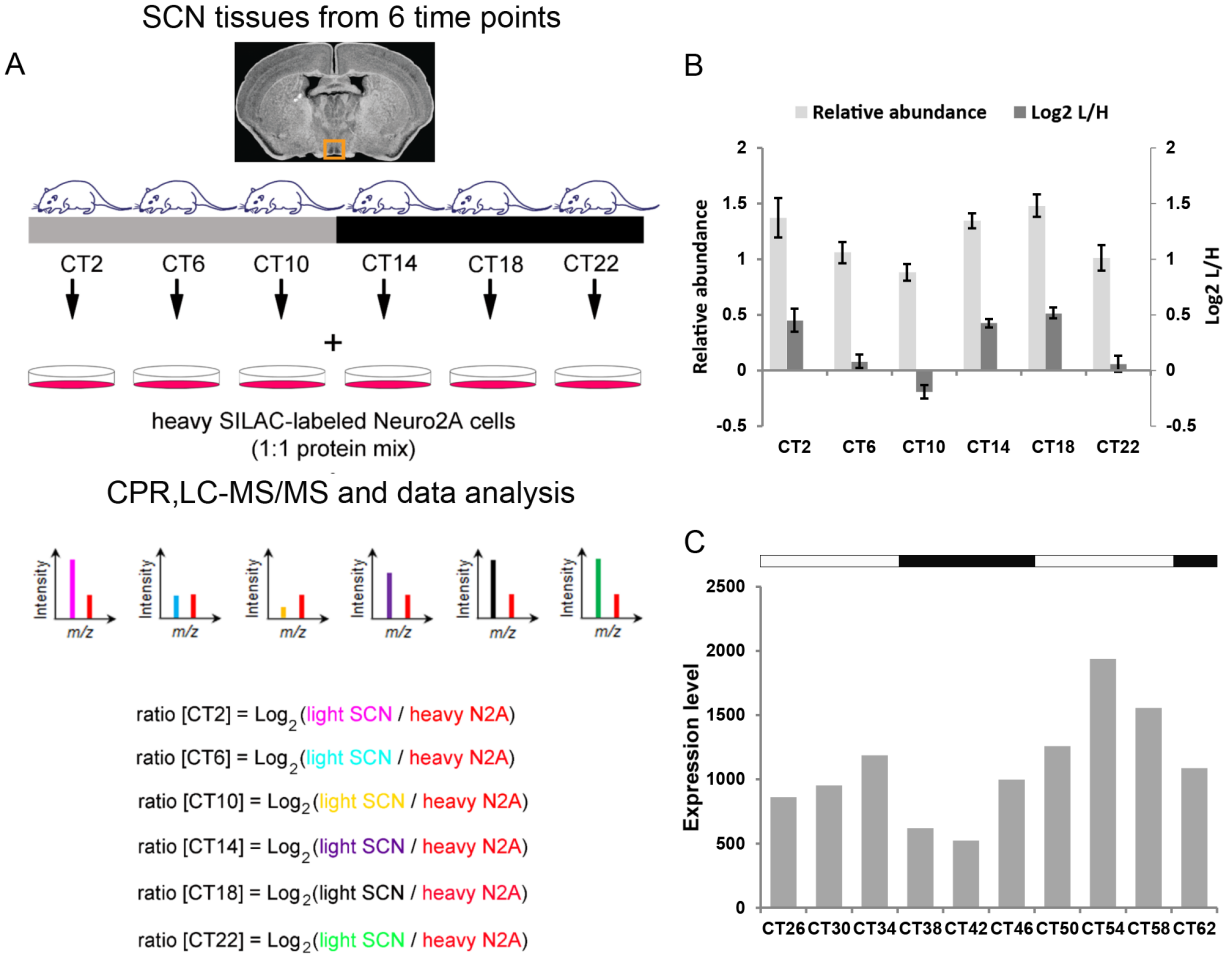
Proteomic analysis of UBR4 in the murine SCN. (**A**) Schematic overview of the centrifugal proteomic reactor (CPR) coupled with SILAC-based quantification of the murine SCN proteome. Unlabeled (“light”) protein lysates extracted from the SCN of individual mice (n = 4 per circadian time point) were mixed with equal quantities of protein lysates from “heavy” SILAC-labeled Neuro2A cells. The mixtures were processed by the CPR coupled with HPLC-ESI-MS/MS. Protein abundance in the “light” SCN samples was determined based on relative abundance to the same heavy-labeled peptide in the Neuro2A reference standard. Relative abundance is expressed as the Log_2_ value of the L/H ratio, where L and H represent light and heavy, respectively. A “ratio of Log_2_ ratios” provides information on relative changes in protein expression between two time points. (**B**) Time-of-day-dependent UBR4 expression profile from mass spectrometry analysis. Data are plotted as median values of Log_2_(L/H) ± SEM (dark gray bars). For ease of interpretation, Log_2_(L/H) values were converted to relative abundance values and expressed as mean ± SEM (light gray bars). *p* = 0.013 (1-way ANOVA). (**C**) UBR4 transcript levels in the murine SCN as detected in a gene expression profiling analysis by Panda et. al. [25]. Data were extracted from circadian expression profile database (CircaDB) (http://circadb.org) [41], dataset: Mouse SCN MAS4 Panda 2002 [25], and replotted as expression level as a function of CT. Tissues were harvested from dark-adapted C57BL/6J mice. White and black bars above the graph indicate the expected light and dark phases, respectively, of the previous LD schedule.

To survey the murine SCN proteome for time-of-day-dependent fluctuations in protein expression, we conducted SILAC-based quantitative mass spectrometry of murine SCN tissues harvested at 4 h intervals across the circadian cycle (Fig. 1A) (Chiang et al., manuscript describing mass spectrometry screen was submitted). In our SILAC (stable isotope labeling by amino acids in cell culture) approach [22], Neuro2A (N2A) cells, which were labeled with heavy isotopes of amino acids by culturing in ‘heavy’ growth medium for >10 passages, were used as the spike-in reference standard for the quantification of the unlabeled SCN proteome. Results were compared with a previous proteomic study [20], based on the semi-quantitative spectral counts approach [23], where we analyzed changes in protein expression in the murine SCN 4 h after a CT 15 light pulse. This 4 h interval was originally chosen to capture a broader subset of light-induced proteins, including those whose abundance altered as a result of changes in gene transcription. Out of 441 and 387 proteins whose expression were deemed to be time-of-day-dependent or light-induced in the SCN based on these two studies, respectively, we focused our attention on one protein that was identified in both screens: UBR4. As shown in Tables 1 and 2, 13 and 7 unique peptide sequences derived from UBR4 were detected in the two mass spectrometry screens. From the Tian et al. study [20], the 7 unique peptides were detected in samples from the CT15 light-pulsed cohort only and not from the dark controls (Table 2). From the Chiang et al. study, the expression of UBR4 protein in the murine SCN showed time-of-day-dependent fluctuations that were statistically significant (p<0.05) by ANOVA (Fig. 1B), but did not fit a 24 h rhythmic profile based on JTK_CYCLE analysis [24]. Data extracted from CircaDB (http://bioinf.itmat.upenn.edu/circa/), a public repository of circadianly expressed transcripts in different cell and tissue types, revealed that levels of *ubr4* transcript were rhythmic in the SCN (Fig. 1C) based on a previous gene profiling experiment by Panda et al. [25]. The transcript peaked in the mid subjective day, whereas protein levels, according to mass spectrometry, appeared higher in the early- to mid-subjective night and early day. In summary, our proteomic analyses suggest that UBR4 protein expression in the SCN is time-of-day-dependent and induced by photic stimulation in the early night.

**Table 1:** UBR4-derived peptides identified in the murine SCN analyzed across the circadian cycle (Chiang et al, manuscript describing mass spectrometry screen was submitted).

Peptide Sequence	# of Samples Detected In (max. 24)	m/z	Charge	Score
ALGTLGMTTNEK	1	617.31	2	38.24
DPELFSGLASNILNFITTSMLNSR	5	879.78	3	43.22
GITQNALDYMK	9	626.31	2	49.35
NLLPATLQLIDTYASFTR	9	1018.05	2	205.88
QKEVQMNFLNQLTSVFNPR	5	764.06	3	65.95
QLISAHVLR	1	517.81	2	73.23
QLLQLLTTYIVR	7	729.94	2	123.95
RANTPLYHGFK	5	651.34	2	56.23
SIAVTRPNNLVHFTESK	13	637.34	3	77.22
SLLASLHSSR	23	534.79	2	111.06
SLTGLLSSFVEVESIKR	7	621.34	3	50.84
TEIITFTAMMK	9	642.32	2	65.72
VLGIYTFTKR	15	598.35	2	97.46

**Table 2:** UBR4-derived peptides identified in the SCN of CT 15 light pulse-treated mice [20].

Peptide Sequence	Spectral Counts	m/z	Charge	Mascot Score
APVYLFEQVQHNLLSPPFGWASGSQDSSSR	1	1102.81	3	32
AVWLPGSQTELAIVTADFVK	1	1073.95	2	39
DLHTLDSHVR	2	597.04	2	39
HNDMPIYEAADK	2	702.76	2	66
LEQVSSDEGIGTLAENLLEALR	2	1179.52	2	163
SLLASLHSSR	1	535.58	2	32
TSPADHGGSVGSESGGSAVDSVAGEHSVSGR	1	948.03	3	65

### UBR4 is Expressed Throughout the Rostrocaudal Extent of the Mouse SCN

**Figure 2:**
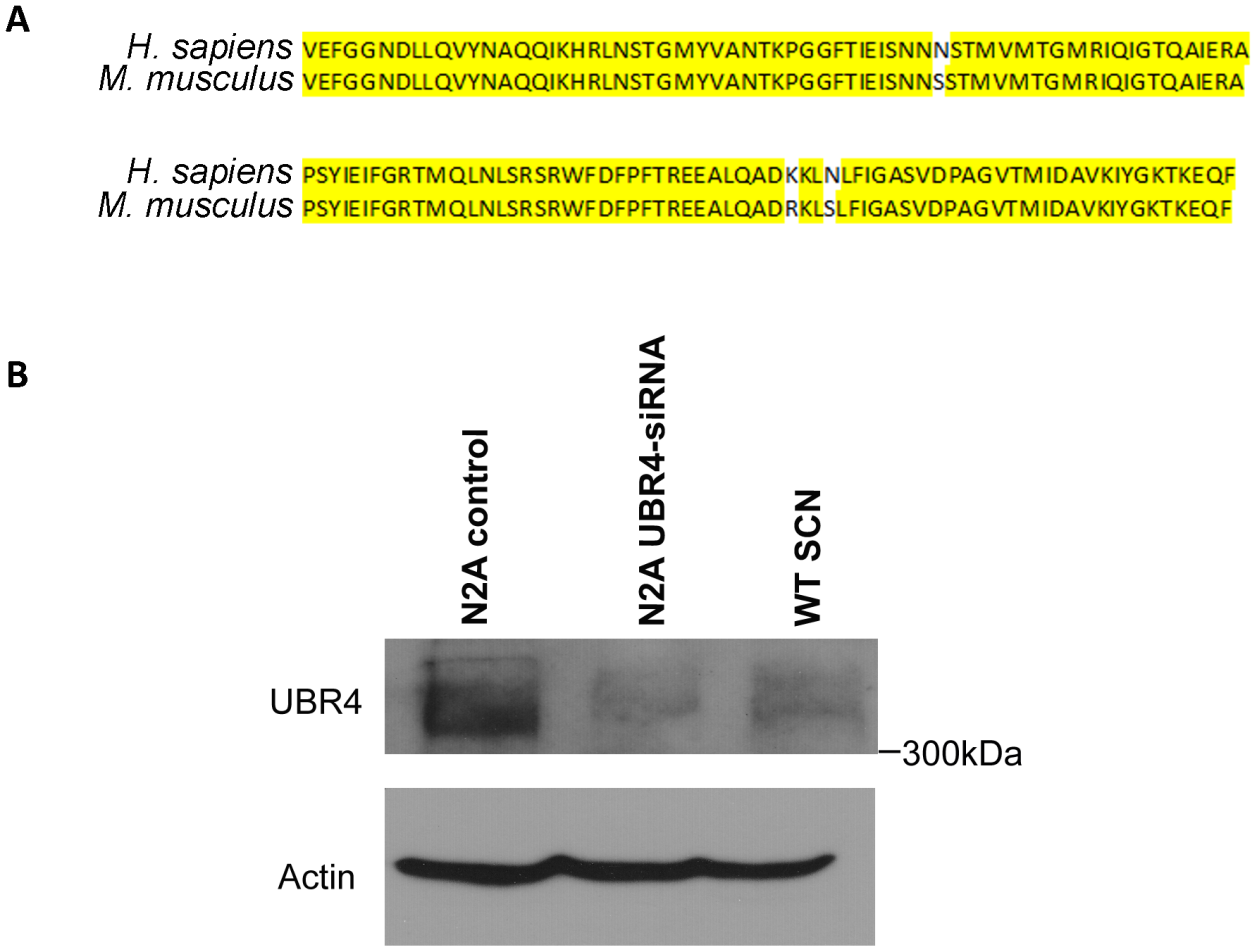
Validating the specificity of the UBR4 antibody employed in the current study. (**A**) The human UBR4 immunogen sequence that was used to generate the rabbit polyclonal UBR4 antibody and its alignment with murine UBR4. Nucleotides that are identical between the two sequences are highlighted in yellow. (**B**) Western blot analysis of UBR4 protein levels in mock-transfected N2A control, N2A cells with siRNA knockdown of UBR4, and C57BL/6J mouse SCN. All lanes expressed a band that was greater than 300 kDa (corresponding to UBR4, predicted size ∼570 kDa), the intensity of which was greatly diminished upon UBR4 siRNA knockdown. Actin was used as the loading control.

In order to examine the spatiotemporal expression of UBR4 in the SCN, we used a commercially available rabbit polyclonal antibody that was developed against a 130-residue epitope of human UBR4 with 98% identity to the murine homolog (Fig. 2A). The full-length UBR4 protein has a predicted size of 570 kDa. By Western blotting, this antibody detected a band of >300 kDa in N2A cell extracts (control) and SCN extracts prepared from wild-type (WT) mice on a C57BL/6J background (Fig. 2B). This band corresponds to UBR4, since signal intensity was greatly diminished in the N2A cell extract that was treated with siRNA targeting UBR4 (Fig. 2B, N2A UBR4-siRNA lane). An additional band at ∼120 kDa was observed in the SCN extract (Fig. S1), although it is unclear whether or not this is a non-specific protein or one of the several predicted splice variants of the murine *ubr4* gene. Based on these results, we conclude that this antibody selectively recognizes UBR4 in the murine SCN.

**Figure 3:**
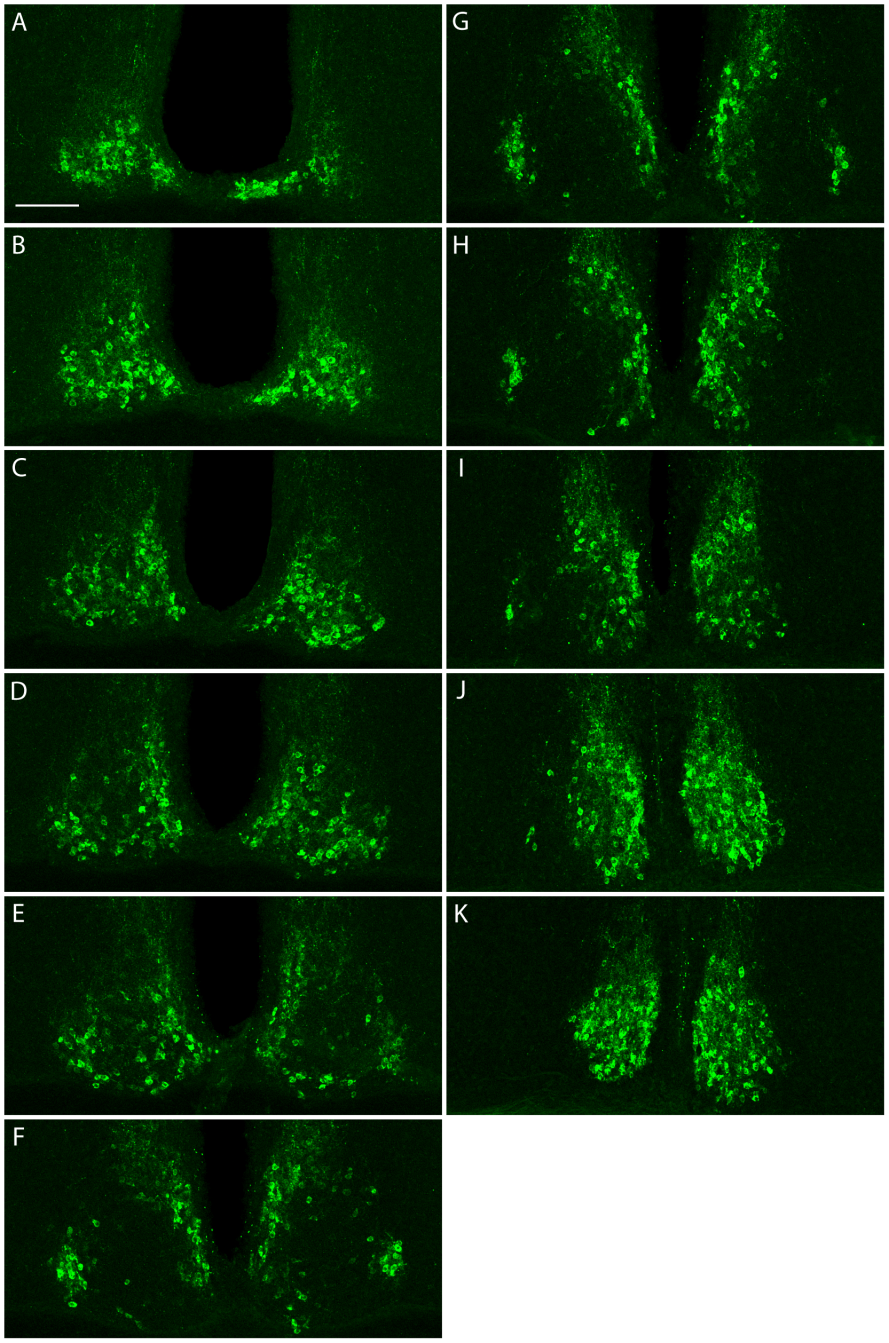
UBR4 is expressed throughout the rostrocaudal extent of the murine SCN. (**A–K**) Sections spanning the entire rostrocaudal extent of the SCN of a mouse that had received a light pulse (15 min, 100 lux) at CT 14 and killed 4 h later were processed for UBR4 expression by indirect immunofluorescence. Sections are arranged serially from the most rostral (A) to the most caudal (K).

Using this antibody, we visualized UBR4 expression in the murine SCN by immunofluorescence staining. Tissues spanning the entire rostrocaudal axis of the SCN of a C57BL/6J mouse that had been light-pulsed (LP: 15 min, 100 lux) at CT 14 and killed 4 h post-LP were selected for processing. As illustrated in Fig. 3, UBR4 is expressed throughout the rostrocaudal extent of the SCN, localizing primarily to the SCN shell (Fig. 3, panels F–H). Consistent with previous reports [26], UBR4 immunoreactivity is predominantly cytoplasmic. These results confirm the presence of UBR4 in the murine SCN.

### Effects of Time-of-Day and Acute Light Exposure on UBR4 Expression in the Murine SCN

**Figure 4:**
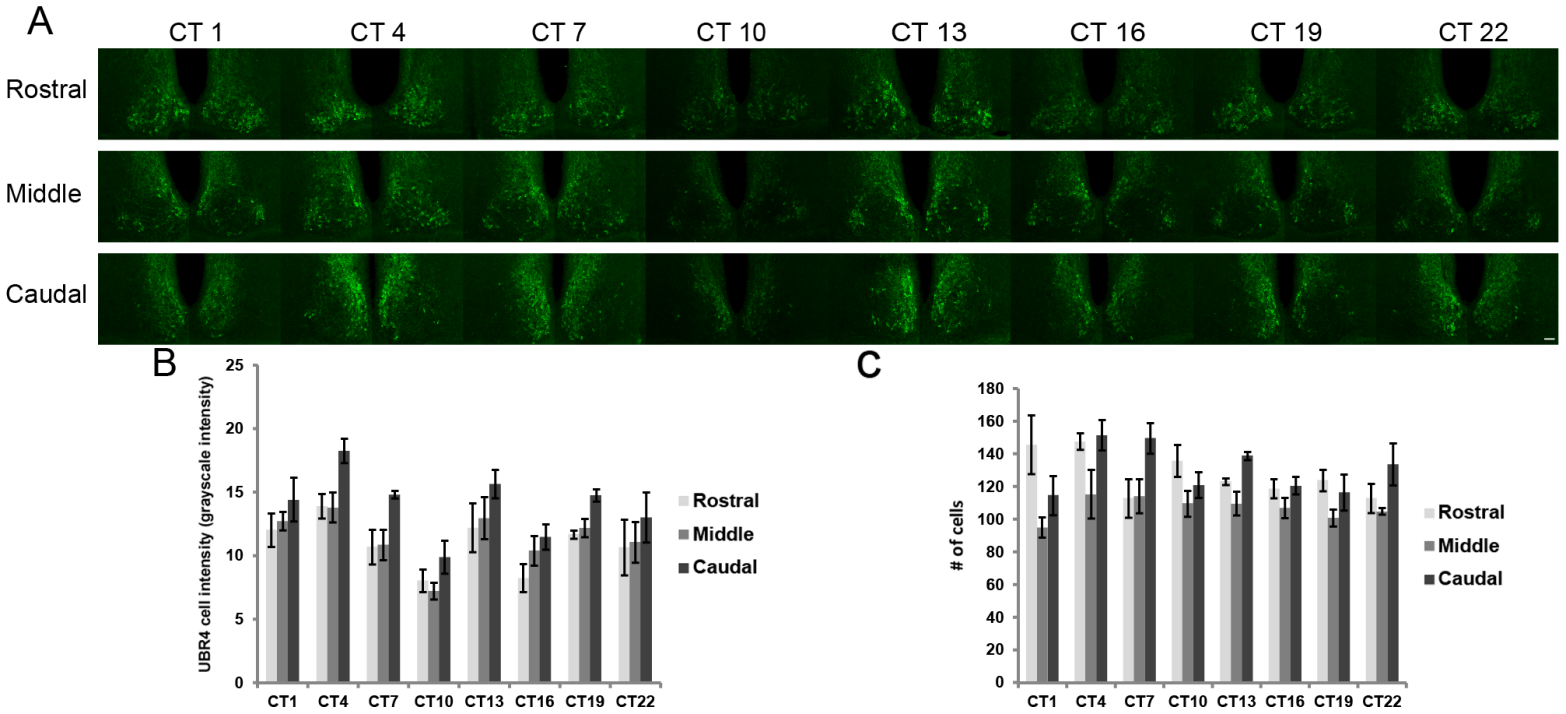
UBR4 in the SCN: time-of-day-dependent expression profile. (**A**) Representative micrographs showing the temporal and spatial expression profile of UBR4 in the SCN. Mice were dark adapted for 2 consecutive days prior to tissue harvest at the indicated circadian times. (**B**) Quantification of mean UBR4 intensity in individual cells in different sections as a function of circadian time. Values were normalized to background staining. *y*-axis represents mean intensity of UBR4 staining in individual cells in grayscale intensity units. n = 4 animals per time point. (**C**) Quantification of the number of UBR4-expressing cells in different SCN sections as a function of circadian time. *y*-axis represents number of counted UBR4-positive cells. n = 4 animals per time point. (Scale bar  = 50 µm).

To confirm our MS data and to further characterize the expression of UBR4 in the SCN, we asked whether UBR4 levels varied across the circadian cycle and in response to photic stimulation applied at different times of day. Following 2 days of dark adaptation, mice were killed at 3 h intervals across the 24 h cycle. As shown in Figs. 4A and B, mean intensity of UBR4 immunofluorescence in individual SCN cells fluctuates as a function of time-of-day. One-way ANOVA-F revealed a significant effect of time on UBR4 signal intensity in individual cells in the middle (m) and caudal (c) sections of the SCN (F_7,24_ = 3.04(m) and 4.43(c), p<0.05), but not the rostral section (F_7,24_ = 2.10, p>0.05). UBR4 expression decreases throughout the day and reaches the nadir at CT10 before increasing again shortly after subjective dusk (CT 13) (Table S1). However, the total number of UBR4-expressing cells in the rostral, middle and caudal SCN does not fluctuate according to time-of-day (F_7,24_ = 1.77(r), 0.624(m), 2.33(c), p>0.05 (r,m,c)) (Fig. 4C).

**Figure 5:**
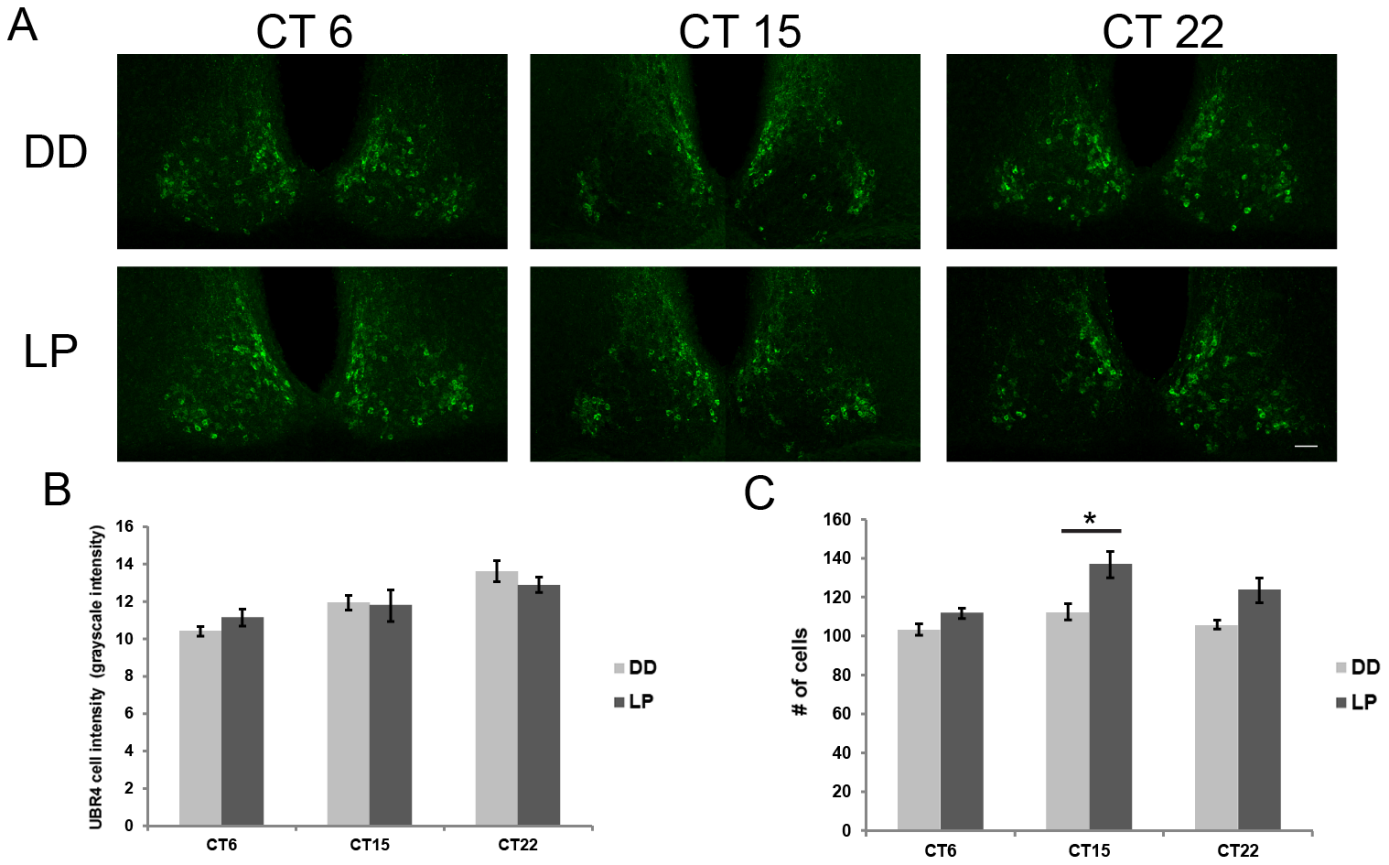
Light inducibility of UBR4 in the SCN depends on time-of-day. (A) Representative micrographs illustrating the expression of UBR4 in the rostral-middle section of the SCN following a light pulse. Light pulses (LP) (15 min, 100 lux) were administered at CT 6, 15 and 22 and tissues were harvested 4 h later. Dark (DD) controls were killed at the same time without prior light treatment. (**B**) Quantification of mean UBR4 intensity in individual cells in the rostral-middle section of the SCN after a light pulse given at the indicated time. Values were normalized to background staining. *y*-axis represents mean intensity of UBR4 staining in individual cells in grayscale intensity units. *x*-axis represents the CT when the light pulse was administered. (**C**) Quantification of the number of UBR4-expressing cells in the rostral-middle section of the SCN after a light pulse given at the indicated time. *y*-axis represents number of counted UBR4-positive cells. n = 4 animals per time point. *p<0.05 LP vs. DD control. (Scale bar  = 50 µm).

Separate cohorts of mice were dark adapted for 2 days prior to receiving a light pulse (15 min, 100 lux) at CT 6, 15 or 22 (Fig. 5A). Tissues were harvested 4 h post-LP and compared with respective dark (DD) controls killed at the same time. Mean UBR4 immunofluorescence intensity in individual SCN cells was not significantly different between LP- and DD-treated mice at the three time points tested (F_1,18_ = 0.016, p>0.05) (Fig. 5B), although there was a significant time effect (F_2,18_ = 11.33, p<0.05) as expected based on time-of-day-dependent variation. However, the total number of UBR4-expressing cells shows a significant effect of time and light treatment (F_2,18_ = 6.97 (time), F_1,18_ = 20.661 (treatment), p<0.05 (light and treatment)), where photic stimulation in the early subjective night (CT 15) significantly increases the number of UBR4-positive cells in the middle SCN sections (t_(6)_ = 3.09, p<0.05) (Fig. 5C). This effect was not observed following a light pulse in the mid subjective day (CT 6) or late subjective night (CT 22) (t_(6)_ = 2.00 (CT 6), 2.67 (CT 22), p>0.05) (Fig. 5C). Closer inspection of the CT 15 LP cohort revealed that the increase in UBR4-immunoreactive cells is observed primarily in the middle SCN sections (t_(6)_ = 3.06, p<0.05), and not in the rostral and caudal SCN sections (t_(6)_ = 1.62 (r), 0.19 (c), p>0.05) (Fig. S2C). Collectively, the data show that UBR4 expression in the SCN is both time-of-day-dependent and regulated by early night light exposure.

### UBR4 Expression Co-Localizes with Arginine Vasopressin (AVP) in the SCN

**Figure 6:**
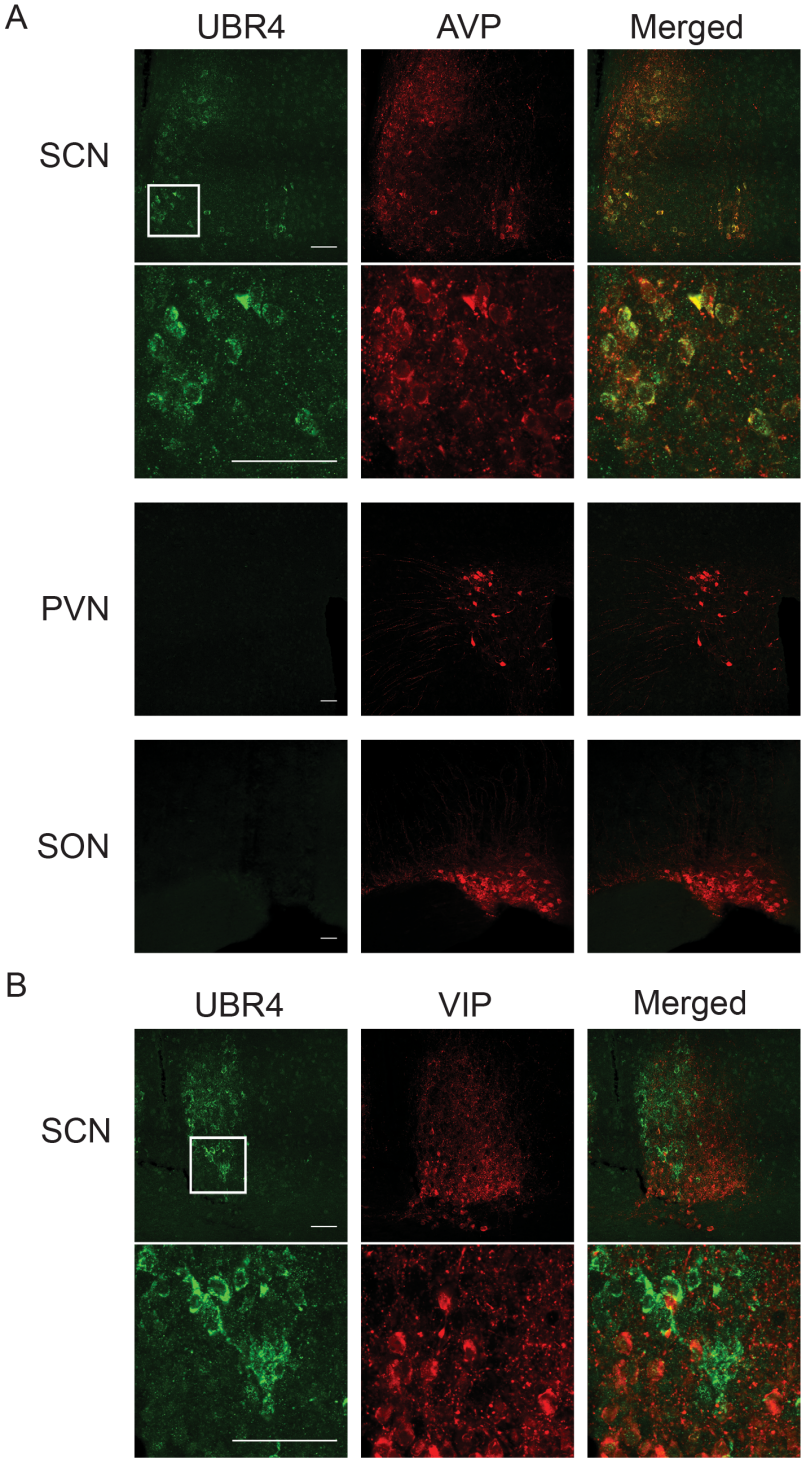
UBR4 is expressed in AVP-positive neurons in the SCN. (**A**) Expression of UBR4 (left-most column) and AVP (middle column) in the SCN, paraventricular nucleus (PVN) and supraoptic nucleus (SON) were assessed by immunofluorescence staining. The right-most column shows the merged image indicating colocalized expression. UBR4 is expressed in AVP-positive cells of the SCN but was not detected in AVP-positive cells of the PVN or SON. (**B**) Expression of UBR4 (left-most column) and VIP (middle column) in the SCN. UBR4 is not expressed in VIP-positive cells in the SCN. For (A) and (B), the boxed regions of the SCN are shown in higher magnification in the lower panels. (Scale bar  = 50 µm).

The SCN is a heterogeneous network of neurochemically distinct cells serving different but interconnected functions. In particular, the two major subdivisions of the SCN, the shell and the core, are delineated by the expression of the neuropeptides arginine vasopressin (AVP) and vasoactive intestinal peptide (VIP), respectively. Whereas circadian rhythms of clock gene expression are most pronounced in the SCN shell, it is the SCN core that first receives light information through direct retinal innervation. The distribution of UBR4 in the SCN shell (Fig. 3F–H) suggests strongly that it is expressed in AVP neurons of the SCN. To confirm this, we performed double immunofluorescence labeling to visualize the extent of colocalization between UBR4 and AVP or VIP. As shown in Fig. 6, UBR4 was detected in AVP- but not VIP-expressing cells in the murine SCN. This colocalization with AVP was detected in sections from rostral, middle and caudal SCN (Fig. S3). Virtually all AVP-positive cells in the SCN also expressed UBR4, and vice versa. This near-perfect colocalization is not the result of cross-reactivity of our primary or secondary antibodies, since UBR4 was not detectable in either the paraventricular nucleus (PVN) or the supraoptic nucleus (SON), two hypothalamic structures that exhibit high expression of AVP (Figs. 6 and S4). We therefore conclude that UBR4 is specifically expressed within AVPergic cells of the murine SCN.

## Discussion

In this study, we established the temporal and spatial expression of a novel E3 ligase component, UBR4, in the murine SCN. Both mass spectrometry and antibody-based detection of *in situ* protein expression revealed the presence of UBR4 in the SCN, fluctuating in a time-of-day-dependent fashion throughout the 24-h day/night cycle. Light in the early subjective night triggered an increase in the number of UBR4-positive cells in the SCN. UBR4 was detected throughout the rostrocaudal extent of the SCN and only in AVP-secreting neurons of the SCN shell compartment. The colocalization of UBR4 and AVP expression was near-perfect. The potential cellular consequences of UBR4 expression in AVPergic cells of the SCN, in a time-of-day- and light-dependent manner, are discussed further below.

UBR4 belongs to the UBR box protein family of E3 ubiquitin ligases, or N-recognins, that functions in the N-end rule degradation pathway [27]. The N-end rule pathway selectively ubiquitinates substrate proteins based on the presence of specific destabilizing residues, or N-degrons, in their N-termini [27]. At present, 7 UBR box proteins (UBR1-7) have been identified in mammals, but their physiological functions remain enigmatic. Previous to our study, the only other report implicating a UBR protein in circadian rhythm regulation came from a study of Neurospora UBR1, which controls the turnover of a morning-expressed transcriptional repressor to produce evening-specific expression of clock-controlled genes [28].

Given the observation that UBR4 is expressed in AVPergic neurons in the SCN, with cellular levels varying as a function of time-of-day, our study suggests a potential role of UBR4 as a modulator of circadian clock timing processes. The SCN is anatomically subdivided into the core, which receives both direct and indirect retinal inputs, and the shell, which is densely innervated by afferents from the core subcompartment as well as from various hypothalamic and limbic structures [29]. The SCN shell and core are also defined neurochemically by the expression of AVP and VIP, respectively. Although core clock proteins are expressed within cells of both the SCN shell and core, it is the shell that exhibits the more robust cycling of PER proteins [30]. Thus, the SCN shell is contemporarily viewed as the “rhythmic” compartment, whereas the core is viewed as the site of photic (and non-photic) integration. Based on the distribution and pattern of UBR4 expression, one possibility is that UBR4 is acting within AVPergic neurons to influence clock timing mechanisms, either directly or indirectly. It is interesting to note that UBR4 levels in the SCN gradually decline throughout the subjective day, when levels of PER1 and PER2 rise in the cytoplasm. Conversely, nuclear levels of PER1 and PER2 peak in the early subjective night; this coincides with a sharp rise in UBR4 expression in the cytoplasm from its nadir at CT 10. One hypothesis that is consistent with these observations is that UBR4 is directly regulating the turnover of a cytoplasmic protein that can influence PER accumulation in the cytoplasm during its rising phase, but might also translocate to the nucleus to affect nuclear PER clearance during its declining phase. Numerous studies have shown that the turnover of PER proteins is exquisitely regulated by components of the ubiquitin proteasome pathway but also by post-translational events such as phosphorylation and acetylation [16], [17], [31], [32].

Light in the subjective night triggers robust and rapid activation of various signal transduction pathways and immediate early genes in the SCN core [9], [33]. These light-evoked events can be communicated to the SCN shell via connecting efferent projections, coordinating the phase re-alignment of these two compartments [29]. Whereas light induces PER1 expression in the SCN core during both the early and late subjective night, PER2 induction is restricted to the early night, exhibits delayed kinetics and is most prominent in the SCN shell [34]. In this regard, light-induced UBR4 expression broadly mirrors the behavior of PER2, in that induction is limited to photic stimulation in the early night and is restricted to the AVPergic cells of the shell subregion, suggesting that the mechanisms which regulate UBR4 and PER2 expression after an early night light pulse may be interconnected. More specifically, early night light exposure increases the number of UBR4-positive cells in the SCN by ∼20%, but does not alter mean levels of UBR4 within each cell. Yan and Silver [34] have suggested that PER2 induction in the shell underlies behavioral phase delays. If, in fact, light-recruited UBR4-immunoreactive cells are the same cells with PER2 induction, one might postulate that UBR4 is influencing the abundance of PER2 in this small subset of AVPergic cells, which in turn would affect light-induced phase delays.

The perfect colocalization of AVP and UBR4 in the SCN suggests that their expression may be mechanistically interconnected. One possible scenario is that UBR4 expression is regulated by AVP signaling. Expression of the vasopressin gene is clock-regulated [35], and both transcript abundance and *in vitro* SCN-AVP release exhibit a circadian rhythm that peaks in the early to mid subjective day [35], [36], preceding the peak in *ubr4* mRNA expression (Fig. 1C). AVP acts primarily on V1a and V1b receptors, the former of which is expressed throughout the SCN, in both the shell and core compartments [37]. It is possible that AVP acting on cognate receptors expressed by shell SCN neurons triggers the transcription of UBR4. A separate study in rat kidney showed that AVP stimulation altered UBR4 expression in this tissue [38]. Additional mechanisms, including miRNAs and translational regulation, likely contribute to further defining the expression of UBR4 protein in the SCN shell, especially given the weak correspondence between the expression profiles of the transcript and the protein. Lastly, given that AVP and its receptors have been shown to contribute to SCN cellular synchrony [39], [40], UBR4, if it is a downstream mediator of AVP signaling, may have an additional role in maintaining phase coherence between SCN core and shell neurons after a phase-delaying light pulse.

In summary, the current study identifies a novel E3 ubiquitin ligase component, UBR4, in the SCN, and provides the basis for future work examining the role of this protein in the regulation of SCN clock timing processes. Our observations raise the possibility that UBR4 participates in core timing mechanisms, photic entrainment, and cellular synchronization within the SCN. Ongoing efforts in our lab are aimed at identifying the physiological targets of UBR4 in the SCN and the impact of reduced UBR4 expression on circadian rhythms.

## Supporting Information

Figure S1 **Whole gel image of UBR4 western blot.** The middle panel shows the whole gel image of UBR4 western blot as depicted in **Fig. 2B** **.** The predicted size of full-length UBR4 is 570 kDa. A band of >300 kDa was observed in all lanes (mock-transfected N2A control, N2A cells treated with UBR4-siRNA, and SCN from wild-type C57BL/6J (WT) mice). This band (arrow) corresponds to UBR4, since UBR4 siRNA-mediated knockdown greatly diminished its signal intensity. Two strong, non-specific bands between 130 and 180 kDa were observed in the N2A samples but not in the SCN samples. An additional band at ∼120 kDa was detected in SCN extracts. Top panel (same as **Fig 2B**) was a re-exposure of the same membrane with only the upper portion of the whole gel (middle panel). Actin (bottom panel) was used as the loading control.
(TIF)

Figure S2 **UBR4 expression along the rostrocaudal extent of the SCN following a CT 15 light pulse.** (**A**) Representative photomicrographs illustrating UBR4 immunoreactivity in the rostral, middle and caudal SCN following a CT15 light pulse (LP). LP and dark (DD) controls were killed at CT19. (**B**) Quantification of mean UBR4 immunofluorescence intensity in individual cells in different sections of the SCN 4 h after a CT15 light pulse. *y*-axis represents mean intensity of UBR4 staining in individual cells in grayscale intensity units. (**C**) Quantification of the number of UBR4-expressing cells in different sections of the SCN after a CT15 light pulse. *y*-axis represents the number of counted UBR4-positive cells. n = 4 animals per time point. *p<0.05 LP vs. DD control. (Scale bar  = 50 µm).
(TIF)

Figure S3 **Colocalization of UBR4 and AVP throughout the rostrocaudal extent of the SCN.** Expression of UBR4 (left-most column) and AVP (middle column) were assessed by immunofluorescence staining in sections from rostral, middle and caudal portions of the SCN. The right-most column shows the merged image indicating colocalized expression. UBR4 is expressed in AVP-positive cells throughout the entire extent of the SCN. (Scale bar  = 50 µm).
(TIF)

Figure S4 **Localization of the SCN, SON and PVN in coronal mouse brain sections.** Coronal brain tissue sections containing the rostral, middle and caudal portions of the SCN were stained with an AVP-specific antibody. The SCN is outlined (dashed line) in all sections. The locations of the SON and PVN are also labeled. (Scale bar  = 100 µm).
(TIF)

Table S1 **Post Hoc Analysis (LSD) for Mean UBR4 Cell Intensity Across the Circadian Cycle.**
(DOCX)

Text S1 **Supplemental methods and references.**
(DOCX)

## References

[1] Reppert SM, Weaver DR (2002) Coordination of circadian timing in mammals. Nature 418: 935–941. 12198538 10.1038/nature00965

[2] Moore RY, Eichler VB (1972) Loss of a circadian adrenal corticosterone rhythm following suprachiasmatic lesions in the rat. Brain Res 42: 201–206. 5047187 10.1016/0006-8993(72)90054-6

[3] Stephan FK, Zucker I (1972) Circadian-Rhythms in Drinking Behavior and Locomotor Activity of Rats Are Eliminated by Hypothalamic-Lesions. Proc Natl Acad Sci U S A 69: 1583–1586. 4556464 10.1073/pnas.69.6.1583 PMC426753

[4] Lowrey PL, Takahashi JS (2011) Genetics of circadian rhythms in mammalian model organisms. Adv Genet 74: 175–230. 21924978 10.1016/B978-0-12-387690-4.00006-4 PMC3709251

[5] Gallego M, Virshup DM (2007) Post-translational modifications regulate the ticking of the circadian clock. Nat Rev Mol Cell Biol 8: 139–148. 17245414 10.1038/nrm2106

[6] Golombek DA, Rosenstein RE (2010) Physiology of Circadian Entrainment. Physiol Rev 90: 1063–1102. 20664079 10.1152/physrev.00009.2009

[7] Moore RY, Lenn NJ (1972) A retinohypothalamic projection in the rat. J Comp Neurol 146: 1–14. 4116104 10.1002/cne.901460102

[8] Johnson RF, Moore RY, Morin LP (1988) Loss of entrainment and anatomical plasticity after lesions of the hamster retinohypothalamic tract. Brain Res 460: 297–313. 2465060 10.1016/0006-8993(88)90374-5

[9] Obrietan K, Impey S, Smith D, Athos J, Storm DR (1999) Circadian regulation of cAMP response element-mediated gene expression in the suprachiasmatic nuclei. J Biol Chem 274: 17748–17756. 10364217 10.1074/jbc.274.25.17748

[10] Haas AL, Siepmann TJ (1997) Pathways of ubiquitin conjugation. FASEB J 11: 1257–1268. 9409544 10.1096/fasebj.11.14.9409544

[11] Busino L, Bassermann F, Maiolica A, Lee C, Nolan PM, et al (2007) SCFFbxl3 controls the oscillation of the circadian clock by directing the degradation of cryptochrome proteins. Science 316: 900–904. 17463251 10.1126/science.1141194

[12] Godinho SI, Maywood ES, Shaw L, Tucci V, Barnard AR, et al (2007) The after-hours mutant reveals a role for Fbxl3 in determining mammalian circadian period. Science 316: 897–900. 17463252 10.1126/science.1141138

[13] Siepka SM, Yoo SH, Park J, Song WM, Kumar V, et al (2007) Circadian mutant overtime reveals F-box protein FBXL3 regulation of cryptochrome and period gene expression. Cell 129: 1011–1023. 17462724 10.1016/j.cell.2007.04.030 PMC3762874

[14] Hirano A, Yumimoto K, Tsunematsu R, Matsumoto M, Oyama M, et al (2013) FBXL21 Regulates Oscillation of the Circadian Clock through Ubiquitination and Stabilization of Cryptochromes. Cell 152: 1106–1118. 23452856 10.1016/j.cell.2013.01.054

[15] Yoo SH, Mohawk JA, Siepka SM, Shan YL, Huh SK, et al (2013) Competing E3 Ubiquitin Ligases Govern Circadian Periodicity by Degradation of CRY in Nucleus and Cytoplasm. Cell 152: 1091–1105. 23452855 10.1016/j.cell.2013.01.055 PMC3694781

[16] Ohsaki K, Oishi K, Kozono Y, Nakayama K, Nakayama KI, et al (2008) The role of {beta}-TrCP1 and {beta}-TrCP2 in circadian rhythm generation by mediating degradation of clock protein PER2. J Biochem 144: 609–618. 18782782 10.1093/jb/mvn112

[17] Reischl S, Vanselow K, Westermark PO, Thierfelder N, Maier B, et al (2007) beta-TrCP1-mediated degradation of PERIOD2 is essential for circadian dynamics. J Biol Rhythms 22: 375–386. 17876059 10.1177/0748730407303926

[18] Naidoo N, Song W, Hunter-Ensor M, Sehgal A (1999) A role for the proteasome in the light response of the timeless clock protein. Science 285: 1737–1741. 10481010 10.1126/science.285.5434.1737

[19] Koh K, Zheng XZ, Sehgal A (2006) JETLAG resets the Drosophila circadian clock by promoting light-induced degradation of TIMELESS. Science 312: 1809–1812. 16794082 10.1126/science.1124951 PMC2767177

[20] Tian RJ, Alvarez-Saavedra M, Cheng HYM, Figeys D (2011) Uncovering the Proteome Response of the Master Circadian Clock to Light Using an AutoProteome System. Mol Cell Proteomics 10: M110.007252. 10.1074/mcp.M110.007252 PMC3226397 21859948

[21] Tasaki T, Mulder LCF, Iwamatsu A, Lee MJ, Davydov IV, et al (2005) A family of mammalian E3 ubiquitin ligases that contain the UBR box motif and recognize N-degrons. Mol Cell Biol 25: 7120–7136. 16055722 10.1128/MCB.25.16.7120-7136.2005 PMC1190250

[22] Ishihama Y, Sato T, Tabata T, Miyamoto N, Sagane K, et al (2005) Quantitative mouse brain proteomics using culture-derived isotope tags as internal standards. Nat Biotechnol 23: 617–621. 15834404 10.1038/nbt1086

[23] Arike L, Peil L (2014) Spectral counting label-free proteomics. Methods Mol Biol 1156: 213–222. 24791991 10.1007/978-1-4939-0685-7_14

[24] Hughes ME, Hogenesch JB, Kornacker K (2010) JTK_CYCLE: an efficient nonparametric algorithm for detecting rhythmic components in genome-scale data sets. J Biol Rhythms 25: 372–380. 20876817 10.1177/0748730410379711 PMC3119870

[25] Panda S, Antoch MP, Miller BH, Su AI, Schook AB, et al (2002) Coordinated transcription of key pathways in the mouse by the circadian clock. Cell 109: 307–320. 12015981 10.1016/s0092-8674(02)00722-5

[26] Nakatani Y, Konishi H, Vassilev A, Kurooka H, Ishiguro K, et al (2005) p600, a unique protein required for membrane morphogenesis and cell survival. Proc Natl Acad Sci U S A 102: 15093–15098. 16214886 10.1073/pnas.0507458102 PMC1247991

[27] Sriram SM, Kim BY, Kwon YT (2011) The N-end rule pathway: emerging functions and molecular principles of substrate recognition. Nat Rev Mol Cell Biol 12: 735–747. 22016057 10.1038/nrm3217

[28] Sancar G, Sancar C, Brugger B, Ha N, Sachsenheimer T, et al (2011) A Global Circadian Repressor Controls Antiphasic Expression of Metabolic Genes in Neurospora. Molecular Cell 44: 687–697. 22152473 10.1016/j.molcel.2011.10.019

[29] Abrahamson EE, Moore RY (2001) Suprachiasmatic nucleus in the mouse: retinal innervation, intrinsic organization and efferent projections. Brain Res 916: 172–191. 11597605 10.1016/s0006-8993(01)02890-6

[30] Hamada T, Antle MC, Silver R (2004) Temporal and spatial expression patterns of canonical clock genes and clock-controlled genes in the suprachiasmatic nucleus. Eur J Neurosci 19: 1741–1748. 15078548 10.1111/j.1460-9568.2004.03275.x PMC3275423

[31] Eide EJ, Woolf MF, Kang H, Woolf P, Hurst W, et al (2005) Control of mammalian circadian rhythm by CKIepsilon-regulated proteasome-mediated PER2 degradation. Mol Cell Biol 25: 2795–2807. 15767683 10.1128/MCB.25.7.2795-2807.2005 PMC1061645

[32] Asher G, Gatfield D, Stratmann M, Reinke H, Dibner C, et al (2008) SIRT1 regulates circadian clock gene expression through PER2 deacetylation. Cell 134: 317–328. 18662546 10.1016/j.cell.2008.06.050

[33] Dziema H, Oatis B, Butcher GQ, Yates R, Hoyt KR, et al (2003) The ERK/MAP kinase pathway couples light to immediate-early gene expression in the suprachiasmatic nucleus. Eur J Neurosci 17: 1617–1627. 12752379 10.1046/j.1460-9568.2003.02592.x

[34] Yan L, Silver R (2002) Differential induction and localization of mPer1 and mPer2 during advancing and delaying phase shifts. Eur J Neurosci 16: 1531–1540. 12405967 10.1046/j.1460-9568.2002.02224.x PMC3281755

[35] Jin X, Shearman LP, Weaver DR, Zylka MJ, de Vries GJ, et al (1999) A molecular mechanism regulating rhythmic output from the suprachiasmatic circadian clock. Cell 96: 57–68. 9989497 10.1016/s0092-8674(00)80959-9

[36] van der Veen DR, Mulder EG, Oster H, Gerkema MP, Hut RA (2008) SCN-AVP release of mPer1/mPer2 double-mutant mice in vitro. J Circadian Rhythms 6: 5. 18355404 10.1186/1740-3391-6-5 PMC2277380

[37] Young WS 3rd, Kovács K, Lolait SJ (1993) The diurnal rhythm in vasopressin V1a receptor expression in the suprachiasmatic nucleus is not dependent on vasopressin. Endocrinology 133: 585–590. 8344200 10.1210/endo.133.2.8344200

[38] Lee YJ, Lee JE, Choi HJ, Lim JS, Jung HJ, et al (2011) E3 ubiquitin-protein ligases in rat kidney collecting duct: response to vasopressin stimulation and withdrawal. Am J Physiol Renal Physiol 301: F883–F896. 21734099 10.1152/ajprenal.00117.2011

[39] Maywood ES, Chesham JE, O'Brien JA, Hastings MH (2011) A diversity of paracrine signals sustains molecular circadian cycling in suprachiasmatic nucleus circuits. Proc Natl Acad Sci U S A 108: 14306–14311. 21788520 10.1073/pnas.1101767108 PMC3161534

[40] Yamaguchi Y, Suzuki T, Mizoro Y, Kori H, Okada K, et al (2013) Mice genetically deficient in vasopressin V1a and V1b receptors are resistant to jet lag. Science 342: 85–90. 24092737 10.1126/science.1238599

[41] Pizarro A, Hayer K, Lahens NF, Hogenesch JB (2013) CircaDB: a database of mammalian circadian gene expression profiles. Nucleic Acids Res. 41(Database issue): D1009–D1013. 23180795 10.1093/nar/gks1161 PMC3531170

