# Supplementary figures and images for: Time-of-Day- and Light-Dependent Expression of Ubiquitin Protein Ligase E3 Component N-Recognin 4 (UBR4) in the Suprachiasmatic Nucleus Circadian Clock
Source: PLoS One. 2014 Aug 1;9(8):e103103. doi: 10.1371/journal.pone.0103103 (PMC4118842; doi:10.1371/journal.pone.0103103)

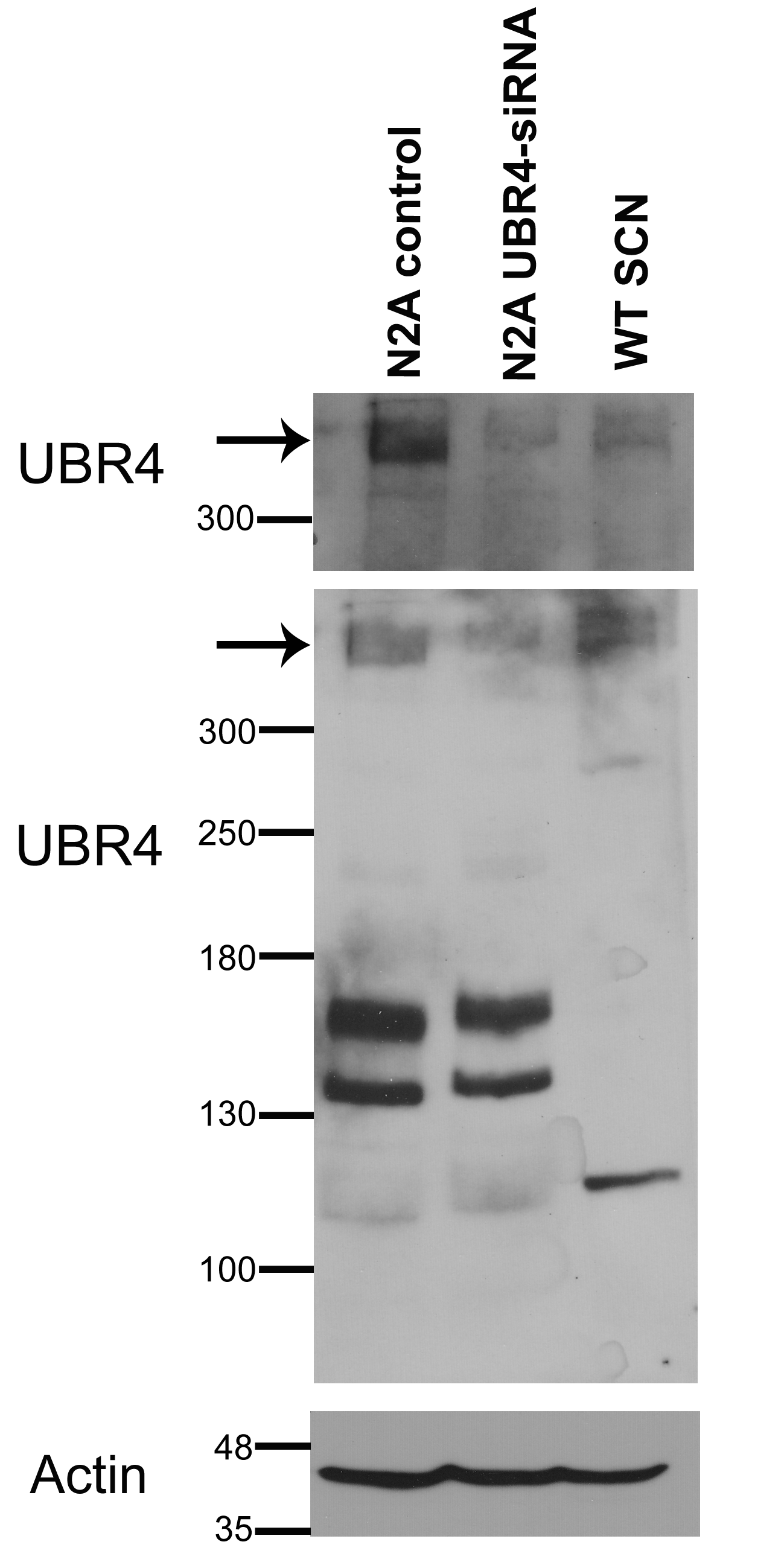

Supplement: Figure S1 — Whole gel image of UBR4 western blot. The middle panel shows the whole gel image of UBR4 western blot as depicted in Fig. 2B . The predicted size of full-length UBR4 is 570 kDa. A band of >300 kDa was observed in all lanes (mock-transfected N2A control, N2A cells treated with UBR4-siRNA, and SCN from wild-type C57BL/6J (WT) mice). This band (arrow) corresponds to UBR4, since UBR4 siRNA-mediated knockdown greatly diminished its signal intensity. Two strong, non-specific bands between 130 and 180 kDa were observed in the N2A samples but not in the SCN samples. An additional band at ∼120 kDa was detected in SCN extracts. Top panel (same as Fig 2B ) was a re-exposure of the same membrane with only the upper portion of the whole gel (middle panel). Actin (bottom panel) was used as the loading control. (TIF) [file pone.0103103.s001.tif]

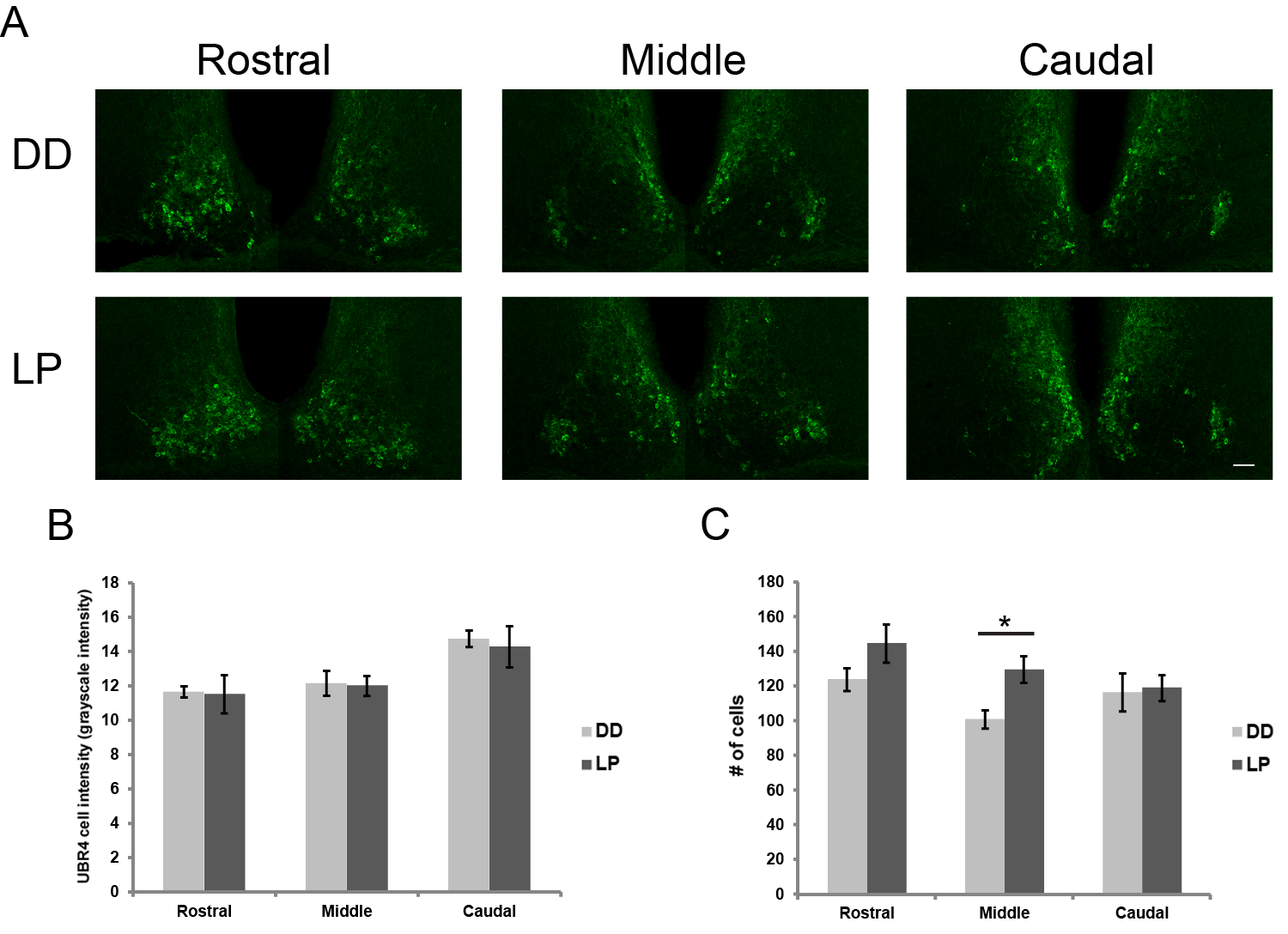

Supplement: Figure S2 — UBR4 expression along the rostrocaudal extent of the SCN following a CT 15 light pulse. (A) Representative photomicrographs illustrating UBR4 immunoreactivity in the rostral, middle and caudal SCN following a CT15 light pulse (LP). LP and dark (DD) controls were killed at CT19. (B) Quantification of mean UBR4 immunofluorescence intensity in individual cells in different sections of the SCN 4 h after a CT15 light pulse. y-axis represents mean intensity of UBR4 staining in individual cells in grayscale intensity units. (C) Quantification of the number of UBR4-expressing cells in different sections of the SCN after a CT15 light pulse. y-axis represents the number of counted UBR4-positive cells. n = 4 animals per time point. *p<0.05 LP vs. DD control. (Scale bar = 50 µm). (TIF) [file pone.0103103.s002.tif]

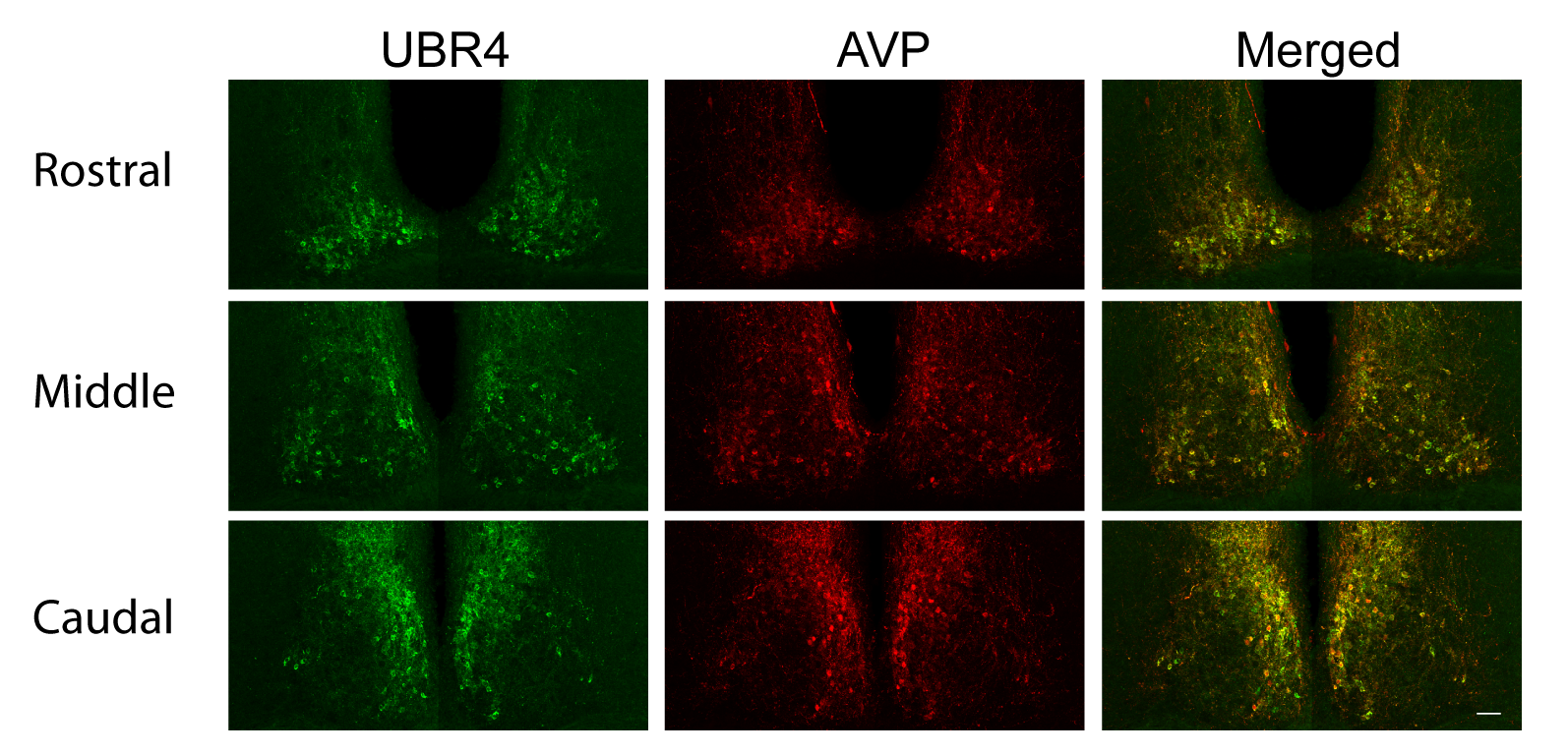

Supplement: Figure S3 — Colocalization of UBR4 and AVP throughout the rostrocaudal extent of the SCN. Expression of UBR4 (left-most column) and AVP (middle column) were assessed by immunofluorescence staining in sections from rostral, middle and caudal portions of the SCN. The right-most column shows the merged image indicating colocalized expression. UBR4 is expressed in AVP-positive cells throughout the entire extent of the SCN. (Scale bar = 50 µm). (TIF) [file pone.0103103.s003.tif]

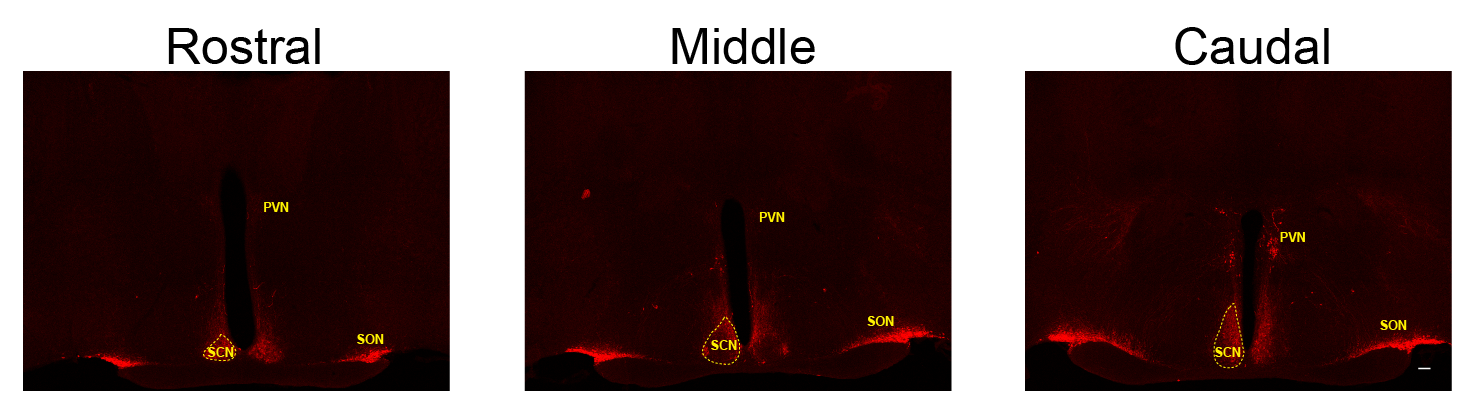

Supplement: Figure S4 — Localization of the SCN, SON and PVN in coronal mouse brain sections. Coronal brain tissue sections containing the rostral, middle and caudal portions of the SCN were stained with an AVP-specific antibody. The SCN is outlined (dashed line) in all sections. The locations of the SON and PVN are also labeled. (Scale bar = 100 µm). (TIF) [file pone.0103103.s004.tif]
