# Supplementary material for: Time-of-Day- and Light-Dependent Expression of Ubiquitin Protein Ligase E3 Component N-Recognin 4 (UBR4) in the Suprachiasmatic Nucleus Circadian Clock
Source: PLoS One. 2014 Aug 1;9(8):e103103. doi: 10.1371/journal.pone.0103103 (PMC4118842; doi:10.1371/journal.pone.0103103)
Supplement: Table S1 — Post Hoc Analysis (LSD) for Mean UBR4 Cell Intensity Across the Circadian Cycle. (DOCX) [file pone.0103103.s005.docx]

**Supporting Table S1: Post hoc analysis (LSD) for mean UBR4 cell intensity across the circadian cycle.**

**Rostral**

|  | CT1 | CT4 | CT7 | CT10 | CT13 | CT16 | CT19 | CT22 |
| --- | --- | --- | --- | --- | --- | --- | --- | --- |
| CT1 | / | --- | --- | --- | --- | --- | --- | --- |
| CT4 | --- | / | --- | 0.006 | --- | 0.008 | --- | --- |
| CT7 | --- | --- | / | --- | --- | --- | --- | --- |
| CT10 | --- | 0.006 | --- | / | 0.044 | --- | --- | --- |
| CT13 | --- | --- | --- | 0.044 | / | --- | --- | --- |
| CT16 | --- | 0.008 | --- | --- | --- | / | --- | --- |
| CT19 | --- | --- | --- | --- | --- | --- | / | --- |
| CT22 | --- | --- | --- | --- | --- | --- | --- | / |

**Middle**

|  | CT1 | CT4 | CT7 | CT10 | CT13 | CT16 | CT19 | CT22 |
| --- | --- | --- | --- | --- | --- | --- | --- | --- |
| CT1 | / | --- | --- | 0.003 | --- | --- | --- | --- |
| CT4 | --- | / | --- | 0.001 | --- | --- | --- | --- |
| CT7 | --- | --- | / | 0.038 | --- | --- | --- | --- |
| CT10 | 0.003 | 0.001 | 0.038 | / | 0.002 | --- | 0.006 | 0.029 |
| CT13 | --- | --- | --- | 0.002 | / | --- | --- | --- |
| CT16 | --- | --- | --- | --- | --- | / | --- | --- |
| CT19 | --- | --- | --- | 0.006 | --- | --- | / | --- |
| CT22 | --- | --- | --- | 0.029 | --- | --- | --- | / |

**Caudal**

|  | CT1 | CT4 | CT7 | CT10 | CT13 | CT16 | CT19 | CT22 |
| --- | --- | --- | --- | --- | --- | --- | --- | --- |
| CT1 | / | 0.036 | --- | 0.016 | --- | --- | --- | --- |
| CT4 | 0.036 | / | --- | <0.001 | --- | 0.001 | --- | 0.006 |
| CT7 | --- | --- | / | 0.009 | --- | --- | --- | --- |
| CT10 | 0.016 | <0.001 | 0.009 | / | 0.003 | --- | 0.010 | --- |
| CT13 | --- | --- | --- | 0.003 | / | 0.024 | --- | --- |
| CT16 | --- | 0.001 | --- | --- | 0.024 | / | --- | --- |
| CT19 | --- | --- | --- | 0.010 | --- | --- | / | --- |
| CT22 | --- | 0.006 | --- | --- | --- | --- | --- | / |

Note: p-values < 0.05 are provided. Dashed lines indicate p>0.05.
