# Supplementary material for: Time-of-Day- and Light-Dependent Expression of Ubiquitin Protein Ligase E3 Component N-Recognin 4 (UBR4) in the Suprachiasmatic Nucleus Circadian Clock
Source: PLoS One. 2014 Aug 1;9(8):e103103. doi: 10.1371/journal.pone.0103103 (PMC4118842; doi:10.1371/journal.pone.0103103)
Supplement: Text S1 — Supplemental methods and references. (DOCX) [file pone.0103103.s006.docx]

**SUPPORTING METHODS**

**SCN Proteomic Analysis in the Chiang et al. Study (Manuscript Describing Mass Spectrometry Screen Was Submitted)**

SCN tissues of individual mice were homogenized in 80 μL lysis buffer (8M urea, 4% CHAPS, 100 mM NH_4_HCO_3_ with fresh proteinase inhibitor mixture) in a 1.5 mL Pellet pestle, and sonicated 3 times for 10 s each with >30 s on ice between each pulse. Protein concentration was determined by the Bradford method. Proteins were processed in a centrifugal proteomic reactor device as previously described [1] with some modifications. Briefly, lysates from SCN tissues and heavy SILAC-labeled Neuro2A cells were mixed 1:1 (30 µg protein each), and vortexed (1 min) vigorously in the presence of 30 µL of SCX slurry and 1.2 mL of 5% formic acid. Samples were centrifuged (16,100 *×g*, 3 min), and SCX bead pellets were washed twice with 1.2 mL 0.1% formic acid. Proteins were reduced by incubating samples in the presence of 20 µL of 150 mM NH_4_HCO_3_, 20 mM DTT (1200 rpm, 56 °C, 15 min), and were subsequently alkylated by the addition of 20 µL of 150 mM NH_4_HCO_3_, 100 mM IAA (room temperature, 15 min in darkness). The reaction was stopped by adding 1.2 mL of 0.1% formic acid supplemented with 6 µg trypsin. Following centrifugation, the SCX bead pellet was resuspended in 40 µL of 1 M NH_4_HCO_3_ and trypsin digested for 4h (37 °C, 1200 rpm). Finally, pH step elution of the peptides from the SCX beads was performed by adding 1.2 ml of 0.1% formic acid pH 2.5, followed by an additional nine pH fractions (pH 3.0, 3.5, 4.0, 4.5, 5.0, 5.5 6.0, 8.0, 12) by subsequent additions of 400 µL 10 mM citric acid/NH_4_OH pH buffers. The fractionated samples were desalted using in-house-made C_18_ desalting cartridges, and desiccated using a Speed-Vac prior to LC-MS measurement.

**LC-MS Measurement**

All resulting peptide fractions were analyzed by HPLC-ESI-MS/MS, which consists of an automated Agilent 1100 micro-HPLC system (Agilent Technologies, Santa Clara, CA) coupled with an LTQ-Orbitrap mass spectrometer (ThermoFisher Scientific, San Jose, CA) equipped with a nano-electrospray interface operated in positive ion mode. Each peptide mixture was reconstituted in 20 µL of 0.5% (v/v) formic acid, and 10 µl was loaded on a 200 μm × 50 mm fritted fused silica pre-column packed in-house with reverse phase Magic C18AQ resins (5μm; 200-Å pore size; Dr. Maisch GmbH, Ammerbuch, Germany). The separation of peptides was performed on an analytical column (75 µm× 10 cm) packed with reverse phase beads (3 μm; 120-Å pore size; Dr. Maisch GmbH, Ammerbuch, Germany). Gradient elution was performed over 75 min from 5-30% acetonitrile (v/v) containing 0.1% formic acid (v/v) at an eluent flow rate of 200 nL/min after in-line flow splitting. The spray voltage was set to +1.8 kV and the temperature of the heated capillary was 200 °C. The instrument method consisted of one full MS scan from 400 to 2000 m/z followed by data-dependent MS/MS scan of the 10 most intense ions, a dynamic exclusion repeat count of 2, and a repeat exclusion duration of 90 s. The full mass was scanned in the Orbitrap analyzer with R = 60,000 (defined at *m/z*=400), and the subsequent MS/MS analysis was performed in the LTQ analyzer. To improve the mass accuracy, all measurements in the Orbitrap mass analyzer were performed with on-the-fly internal recalibration (“Lock Mass”). The charge state rejection function was enabled, and single charge and unassigned charge ions were rejected. All data were recorded with the Xcalibur software (ThermoFisher Scientific, San Jose, CA).

**Database Search and Bioinformatic Analysis**

Raw files were processed and analyzed by MaxQuant, Version 1.3.0.5 against the mouse International Protein Index protein sequence database (IPI Mouse, version 3.75), including commonly observed contaminants. The following parameters were used: cysteine carbamidomethylation was selected as a fixed modification; and the methionine oxidation and protein N-terminal acetylation were set variable modifications. Enzyme specificity was set to trypsin. Up to two missing cleavages of trypsin were allowed. SILAC double labeling (light: K0R0; heavy: K8R10) was set as the search parameter in order to assess the conversion efficiency. The precursor ion mass tolerances were 6 ppm, and fragment ion mass tolerance was 0.8 Da for MS/MS spectra. The false discovery rate (FDR) for peptide and protein was set at 1% and a minimum length of six amino acids was used for peptide identification.

The proteingroup file was imported into Perseus (version 1.3.0.4) for statistical analysis of the data. The raw dataset was filtered to include only proteins with values in at least 12 of 24 MS measurements (or 24 independent SCN samples), resulting in a stringently quantified dataset. One-way ANOVA was used to analyze this stringent dataset for temporal regulation, with p-values < 0.05 indicating statistical significance. JTK_CYCLE algorithm under R language was used to identify proteins that exhibit circadian rhythms with a period of roughly 24h. The values for the ratio L/H normalized of each protein profile from 24 mice were used, and any missing values were replaced with zero prior to JTK_CYCLE analysis. p-values (ADJ.P) less than 0.05 were considered significant.

**SUPPORTING REFERENCES**

1. Zhou H, Wang F, Wang Y, Ning Z, Hou W, et al. (2011) Improved recovery and identification of membrane proteins from rat hepatic cells using a centrifugal proteomic reactor. Mol Cell Proteomics 10: O111.008425.
